# Machine Learning and Clustering Analysis of Class II and III Malocclusions

**DOI:** 10.1002/cre2.70384

**Published:** 2026-06-01

**Authors:** Eva Paddenberg‐Schubert, Kareem Midlej, Sebastian Krohn, Iqbal M. Lone, Osayd Zohud, Obaida Awadi, Samir Masarwa, Aysar Nashef, Christian Kirschneck, Nezar Watted, Peter Proff, Fuad A. Iraqi

**Affiliations:** ^1^ Department of Orthodontics, University Hospital of Regensburg University of Regensburg Regensburg Germany; ^2^ Department of Clinical Microbiology and Immunology, Faculty of Medicine and Health Sciences Tel Aviv University Tel Aviv Israel; ^3^ Center for Dentistry Research and Aesthetics Jatt Israel; ^4^ Department of Oral and Maxillofacial Surgery Meir Medical Center Kfar Saba Israel; ^5^ Faculty of Medicine and Health Sciences Tel‐Aviv University Tel‐Aviv Israel; ^6^ Department of Orthodontics University of Bonn Bonn Germany; ^7^ Gathering for Prosperity Initiative Jatt Israel; ^8^ Department of Orthodontics, Faculty of Dentistry Arab America University Jenin PNA

**Keywords:** congenital, hereditary, malocclusion classification, neonatal diseases, population characteristics, stomatognathic diseases, stomatognathic system

## Abstract

**Objectives:**

This prospective observational study aims to accurately classify individuals as skeletal class II/III by applying several machine‐learning algorithms. Furthermore, using the k‐means clustering analysis deepened our understanding of the characteristics of skeletal class II/III malocclusion (SCIIMO/SCIIIMO) patients.

**Materials and Methods:**

This multicenter study consisted of 379 German orthodontic patients. Following their distribution to SCIIMO (*n* = 208, 54.8%) or SCIIIMO (*n* = 171, 45.1%) by the individualized ANB of Panagiotidis and Witt. Machine‐learning analysis was performed using different models (KNN, RF, LDA, SVM, CART) to find the model with the highest accuracy in correctly classifying skeletal class II/III. After determining the ideal number of clusters using the Elbow approach, k‐means clustering was finally used.

**Results:**

The KNN machine learning model demonstrated the ability of reduced cephalometric parameters to capture much of the same sagittal discriminatory information as the predefined class construct. The results showed that Wits‐appraisal only leads to achieving high accuracy in determining skeletal class (accuracy = 94.72%), while adding SN‐Pg angle improved the accuracy slightly to 95.86%. The clustering analysis showed that three or four clusters were optimal for SCIIMO and SCIIIMO patients and revealed interesting characteristics for each cluster and significance between the differences in all parameters except S‐N and age.

**Conclusions:**

A reduced parameter set that included only the Wits appraisal and SN‐Pg angle enabled a correct classification. In the exploratory clustering analysis, three or four distinct clusters emerged as optimal when analyzing the differences among SCIIMO/SCIIIMO patients, emphasizing the complexity and diversity of malocclusion phenotype.

## 1 Introduction

Various approaches to defining an individual's skeletal class are described in the literature, and differences occur due to the parameter assessed and the kind of norm values needed to interpret the measured parameter. For example, the ANB angle, introduced by Riedel (1952), or the Wits appraisal, established by Jacobson (1975). The average values can be distinguished between empirical and individualized ones. Whereas the first is based on average values, representing a healthy population's mean of a predefined value for a specific parameter, the latter reflects the individually present correlation between different craniofacial structures. Since the first attempt to individualize the average value of ANB for the diagnosis of the skeletal class by Steiner (1953), various techniques have been presented, among which a graphic solution, the harmony box of Segner and Hasund (2003; Segner 1989), and mathematical ones, such as regression equations for the ANB angle (Paddenberg et al. 2023; Panagiotidis and Witt 1977) and the Wits appraisal are frequently applied (Järvinen 1988).

It is mandatory to diagnose the skeletal class of the patient precisely. An individualized plan can be established to improve the efficacy and reduce the undesired side effects of an orthodontic treatment. Besides the routinely used cephalometric analysis already mentioned, genotype analysis has become increasingly popular to identify correlations between specific single‐nucleotide polymorphisms (SNPs) and cephalometric phenotypes (Balkhande et al. 2018; Kirschneck et al. 2022; Milosevic et al. 2022).

Artificial intelligence (AI) is a relatively new method and is intended to assist the practitioner in analyzing images or casts, for example. In dentistry, AI is applied in different fields, including the identification of cephalometric reference points (Schwendicke et al. 2021), cephalometric analysis (Kunz et al. 2020), and assistance in decision‐making (Evangelista et al. 2022). Various machine‐learning models are available, which may differ in their performance and reliability in their specific applications (Bomfim 2024). Therefore, the main aims of this study were to examine the accuracy of comparative machine‐learning models, while using reduced predictor sets such as Wits appraisal, and the SN‐Pg angle, and validating the performance on a strictly unseen data set. Finally, we applied clustering analysis to explore the characteristics of skeletal malocclusion phenotype among the German cohort.

## 2 Material and Methods

### 2.1 Ethical Statement

This observational multi‐center diagnostic study was based on German orthodontic patients treated at different specialist offices. All patients included in the study agreed to participate and signed written consent. Throughout the study, we followed the ethical approval guidelines as well as the Declaration of Helsinki. The study size was determined using the available data collected. In addition, each machine‐learning model was cross‐validated to estimate its performance on unseen data, in terms of correctly classifying.

### 2.2 Cephalometric Analysis

The inclusion criteria applied during recruitment were SCIIMO or SCIIIMO as diagnosed by the definition of Panagiotidis and Witt (1977), pre‐treatment lateral cephalograms available from patients' records, with the possibility of calibration and anamnestic information as evident from the patient's records, including demographic data (age, gender). Concerning the diagnosis of skeletal class, the following criteria were applied:

- SCIIMO: Calculated_ANB > 1,5°
- SCIIIMO: Calculated_ANB < −1,5°
- Calculated_ANB = ANB_measured_ – ANB_individual_
- ANB_individual_ (14) = −35.16 + (0.4 × SNA) + (0.2 × ML‐NSL)

In the present study, slightly different limits of acceptance were applied (±1.5°) than initially proposed by Panagiotidis and Witt (1977) (±1°) to exclude borderline cases from the analysis. Based on the Calculated_ANB, patients were allocated to the group's skeletal classes II and III. Figure S1 and Table S1 explain the relevant cephalometric variables.

### 2.3 Machine‐Learning Models

Machine‐learning models were used to classify individual patients as SCIIMO and SCIIIMO, and the reliability was assessed using kappa. This study included four tiers (A–D). In the first tier (tier A), which was done as a sanity check only, we included the parameters SNA, SNB, and ML‐NSL. In the next tier (B), we included all cephalometric parameters, in addition to gender and age, while excluding the variables which involved in computing the label (ANB, ANBind, Calculated_ANB, SNA, SNB, and ML‐NSL), and in tier C, we included the most important variable as per tier B, and performed the analysis twice, without and with noise. Finally, in tier D, we added the next most important variable, and again here, performed the analysis with and without noise.

K‐nearest neighbor (KNN) assigns an unclassified data point to one of the classes presented and defined in the data set by evaluating those classified points around a new point (Qahaz et al. 2023; Lone et al. 2023b). K refers to the number of points considered to determine the class of the latest unclassified point. The value of *k* was chosen by identifying the KNN model with the highest accuracy: if the input parameter sagittal Wits only was considered to classify skeletal class, *k* equaled 7, whereas in the case Wits appraisal and SN‐Pg angle were used as input variables, the *k*‐value elected was 5.

Another machine‐learning model is the random forest (RF) method, in which the classification results of several independent decision trees are combined to classify a new data point correctly. Each tree decision is made independently of the others by analyzing the parameters presented as input data (Lone et al. 2023a).

The model linear discriminant analysis (LDA) reduces the dimensions of the data set presented into a single dimension and aims to identify a linear relation between the different classes given in the data set. Technically, in this method, the distance between the means of the various data set classes is maximized, whereas the variance within each of the classes presented is minimized (Zohud et al. 2024).

The principle of the machine‐learning model support vector machine (SVM) is the identification of a hyperplane, which is considered a borderline to allocate a new data point to the correct class. Non‐linear input data points are re‐orientated to generate a linear dimension to identify the hyperplane and thereby classify new data points.

Furthermore, the classification and regression trees (CART) model was performed and assessed in terms of its performance in the classification of skeletal classes II/III. This method will assign an unclassified data point to a class by testing several predictive variables in a specific order.

### 2.4 Models Validation Workflow

Before performing any model in this paper, we preprocessed the data with centering and scaling using the R package Caret. Besides, each of the machine‐learning models described above was validated and evaluated for correctly classifying skeletal class II/III by conducting the technique k‐fold cross‐validation using the R package Caret. The *k*‐value chosen for the cross‐validation was 10, and the numbers used were 70% (*n* = 265) of the total study collective. After performing the cross‐validation and getting the mean accuracy results. We examined the best fitting model on unseen data (30% validation data), by running a confusion matrix to compare the actual diagnosis as per the Calculated_ANB, compared to the machine learning classification. Finally, in all novel models, that is, tiers C and D, we examined the models using Gaussian noise to test the practical utility, while ensuring preprocessing.

### 2.5 Clustering

After scaling the data, the clustering algorithm was performed for 3 and 4 clusters, including SCIIMO and SCIIIMO patients. A scatter plot and dendrogram were produced to implement the visualization of the cluster analysis results. We got the optimal cluster number using the Elbow Method (Nainggolan et al. 2019; Yuan and Yang 2019). In addition, we used the Jaccard stability check for *k* = 3 and *k* = 4.

In the next step, we employed k‐means clustering to group patients according to the cephalometric parameters. The k‐means clustering algorithm is one of the commonly used data clustering approaches. The k‐means clustering algorithm receives as input a set of points and k desired centers (cluster representatives). As a result, each set of points has a defined center that they belong to that minimizes the distance to a center for all the possible choices (Wilkin and Huang 2007).

### 2.6 Data Analysis

Intra‐rater and inter‐rater reliability were tested. All cephalogram images were conducted by two trained raters (S.K., E.P.S.) after calibration. Besides, 50 cephalometric images were randomly chosen and analyzed by two independent raters (S.K., E.P.S.). Intra‐rater reliability was assessed by repeating the analysis of the lateral cephalograms by the same investigator with a time interval of at least 2 weeks to avoid bias. The inter‐rater and intra‐rater reliability analysis proved to be almost perfect, indicated by ranges between 0.92 and 0.99, and 0.90 and 0.99, respectively. The reliability between repeated measurements of the two observers, as well as the interrater agreement, was assessed by using Bland–Altman analyses, reporting the confidence intervals of the limits of agreement. Correlation coefficients (Pearson/Spearman) are also reported in order to describe associations of the mentioned measurements (Table 1A, 1B, 1C). Statistical analysis was conducted using the R software platform (https://www.r-project.org/). Results were considered statistically significant and highly significant if *p* < 0.05 and 0.01, respectively.

**Table 1A:** The intrarater reliability calculations of observer 1 for repeated measurements (M1 vs. M2) of cephalometric analyses (1A). Data are presented as mean, standard deviation, and *p* values from normality testing (Shapiro–Wilk). *p*‐values of Shapiro–Wilk‐tests were conducted in order to select the correlation method (Pearson vs. Spearman) for measurements of association (Rho) between the repeated measurements. Furthermore, confidence intervals of limits of agreement acquired from Bland–Altman analyses are shown to further analyze the results about potential systematic deviations.

Parameter	Mean (M1)	Standard deviation (M1)	*p*‐value Shapiro–Wilk‐test (M1)	Mean (M2)	Standard deviation (M2)	*p*‐value Shapiro–Wilk‐test (M2)	Correlation method	Rho	Confidence interval (95%)
SNA [°]	82.00	± 4.16	0.621	81.90	± 4.24	0.267	Pearson	0.989	[−1.063; 1.327]
SNB [°]	78.05	± 3.87	0.683	77.98	± 3.97	0.400	Spearman	0.991	[−0.791; 0.971]
ANB [°]	3.87	± 3.16	0.123	3.82	± 3.19	0.090	Pearson	0.989	[−0.870; 0.980]
Wits [mm]	−0.04	± 4.15	0.206	−0.29	± 4.14	0.535	Pearson	0.950	[−2.311; 2.803]
SN‐Ba [°]	132.67	± 4.84	0.945	132.72	± 4.83	0.643	Pearson	0.977	[−1.909; 1.913]
SN‐Pg [°]	78.84	± 3.92	0.922	78.72	± 3.99	0.843	Pearson	0.992	[−0.856; 1.096]
S‐N [mm]	65.97	± 4.19	0.902	66.06	± 4.22	0.688	Pearson	0.993	[−1.019; 0.823]
Go‐Me [mm]	66.46	± 5.41	0.053	66.54	± 5.36	0.078	Pearson	0.980	[−2.206; 2.054]
Pg/NB [mm]	1.38	± 1.41	0.437	1.29	± 1.33	0.213	Pearson	0.958	[−0.709; 0.889]
NL/NSL [°]	7.18	± 3.63	0.952	7.34	± 3.67	0.993	Pearson	0.968	[−1.810; 1.614]
SN/ML [°]	31.32	± 5.76	0.834	31.33	± 5.71	0.861	Pearson	0.993	[−1.373; 1.345]
NL/ML [°]	24.20	± 4.81	0.464	24.13	± 4.64	0.235	Pearson	0.981	[−1.740; 1.880]
PFH/AFH [%]	67.02	± 5.55	0.88	66.95	± 5.48	0.895	Pearson	0.990	[−1.363; 1.523]
Gonial angle [°]	122.11	± 7.64	0.757	122.28	± 7.30	0.864	Pearson	0.984	[−2.895; 2.559]
Facial axis [°]	91.25	± 4.01	0.270	91.18	± 4.04	0.190	Pearson	0.989	[−1.086; 1.234]
+1/NL [°]	70.11	± 10.09	0.258	69.64	± 9.70	0.313	Pearson	0.973	[−4.035; 4.291]
+1/SN [°]	76.99	± 9.81	0.238	76.96	± 9.50	0.813	Pearson	0.984	[−3.459; 3.519]
+1/NA [°]	21.09	± 9.67	0.050	21.26	± 9.29	0.092	Pearson	0.972	[−4.015; 3.667]
+1i/NA [mm]	3.26	± 3.06	0.551	3.25	± 2.91	0.224	Pearson	0.972	[−1.420; 1.424]
−1/MeGo [°]	84.36	± 10.07	0.086	84.64	± 9.59	0.014	Spearman	0.968	[−4.024; 3.760]
−1/NB [°]	24.43	± 7.85	0.173	24.23	± 7.40	0.031	Spearman	0.958	[−3.562; 3.966]
−1i/NB [mm]	4.16	± 2.40	0.297	4.10	± 2.49	0.125	Pearson	0.989	[−0.660; 0.780]
Interincisal angle [°]	130.89	± 15.70	0.058	130.93	± 15.37	0.022	Spearman	0.966	[−5.329; 5.157]

**Table 1B:** Shows the intrarater reliability calculations of observer 2 for repeated measurement (M1 vs. M2) of cephalometric analyses.

Parameter	Mean (M1)	Standard deviation (M1)	*p*‐value Shapiro–Wilk‐test (M1)	Mean (M2)	Standard deviation (M2)	*p*‐value Shapiro–Wilk‐test (M2)	Correlation method	Rho	Confidence interval (95%)
SNA [°]	82.10	± 4.09	0.690	82.03	± 4.06	0.634	Pearson	0.996	[−0.665; 0.805]
SNB [°]	78.29	± 3.86	0.747	78.26	± 3.82	0.702	Pearson	0.997	[−0.553; 0.605]
ANB [°]	3.90	± 3.07	0.036	3.91	± 3.03	0.029	Spearman	0.995	[−0.444; 0.540]
Wits [mm]	−0.20	± 4.01	0.670	−0.18	± 4.04	0.503	Pearson	0.993	[−0.986; 0.938]
SN‐Ba [°]	132.67	± 4.84	0.279	132.72	± 4.83	0.292	Pearson	0.994	[−1.147; 0.819]
SN‐Pg [°]	79.10	± 3.93	0.940	79.07	± 3.93	0.849	Pearson	0.998	[−0.508; 0.576]
S‐N [mm]	65.99	± 3.79	0.683	66.01	± 3.76	0.605	Pearson	0.998	[−0.482; 0.430]
Go‐Me [mm]	67.84	± 5.12	0.039	67.92	± 5.19	0.050	Spearman	0.984	[−1.554; 1.190]
Pg/NB [mm]	1.29	± 1.47	0.539	1.29	± 1.44	0.271	Pearson	0.984	[−0.529; 0.545]
NL/NSL [°]	6.89	± 3.40	0.948	6.93	± 3.45	0.889	Pearson	0.978	[−1.444; 1.352]
SN/ML [°]	31.17	± 5.65	0.676	31.24	± 5.70	0.840	Pearson	0.995	[−1.217; 1.061]
NL/ML [°]	23.99	± 4.62	0.328	24.02	± 4.56	0.423	Pearson	0.990	[−1.298; 1.242]
PFH/AFH [%]	66.81	± 5.06	0.625	66.69	± 5.11	0.837	Pearson	0.993	[−1.029; 1.257]
Gonial angle [°]	121.73	± 7.72	0.570	121.62	± 7.63	0.556	Pearson	0.995	[−1.416; 1.624]
Facial axis [°]	90.79	± 4.17	0.245	90.67	± 4.38	0.108	Pearson	0.930	[−3.059; 3.283]
+1/NL [°]	69.24	± 9.23	0.057	69.11	± 9.25	0.055	Pearson	0.996	[−1.795; 1.479]
+1/SN [°]	76.29	± 9.58	0.171	76.20	± 9.52	0.165	Pearson	0.996	[−1.608; 1.788]
+1/NA [°]	21.60	± 9.30	0.057	21.76	± 9.27	0.055	Pearson	0.995	[−1.795; 1.479]
+1i/NA [mm]	3.36	± 2.86	0.347	3.48	± 2.89	0.259	Pearson	0.991	[−0.875; 0.639]
−1/MeGo [°]	84.05	± 9.53	0.056	83.94	± 9.24	0.015	Spearman	0.973	[−3.512; 3.732]
−1/NB [°]	25.12	± 8.27	0.261	25.30	± 8.02	0.087	Pearson	0.975	[−3.758; 3.410]
−1i/NB [mm]	4.11	± 2.42	0.089	4.02	± 2.20	0.054	Pearson	0.954	[−1.241; 1.605]
Interincisal angle [°]	129.46	± 15.51	0.018	129.19	± 15.53	0.006	Spearman	0.975	[−3.972; 4.536]

**Table 1C:** Shows the interrater reliability of cephalometric analyses of the two observers. Again, *p*‐values of the Shapiro–Wilk tests were utilized in the selection of the correlation method. Confidence intervals of the limits of agreement are also shown in order to further statistically describe the observers' reliability.

Parameter	*p*‐value Shapiro–Wilk‐test (Rater 1)	*p*‐value Shapiro–Wilk‐test (Rater 2)	Correlation method	Rho	Confidence interval (95%)
SNA [°]	0.621	0.690	Pearson	0.993	[−1.135; 0.783]
SNB [°]	0.683	0.747	Pearson	0.996	[−0.878; 0.402]
ANB [°]	0.013	0.036	Spearman	0.986	[−0.674; 0.798]
Wits [mm]	0.206	0.670	Pearson	0.960	[−2.130; 2.442]
SN‐Ba [°]	0.945	0.279	Pearson	0.958	[−1.928; 3.228]
SN‐Pg [°]	0.922	0.940	Pearson	0.997	[−0.901; 0.381]
S‐N [mm]	0.902	0.683	Pearson	0.996	[−0.731; 0.555]
Go‐Me [mm]	0.053	0.039	Spearman	0.981	[−2.792; 0.904]
Pg/NB [mm]	0.437	0.539	Pearson	0.977	[−0.674; 0.614]
NL/NSL [°]	0.952	0.948	Pearson	0.973	[−1.339; 1.767]
SN/ML [°]	0.834	0.676	Pearson	0.992	[−0.994; 1.866]
NL/ML [°]	0.464	0.328	Pearson	0.977	[−1.810; 2.218]
PFH/AFH [%]	0.880	0.625	Pearson	0.989	[−1.654; 1.330]
Gonial angle [°]	0.757	0.570	Pearson	0.986	[−1.666; 2.442]
Facial Axis [°]	0.274	0.245	Pearson	0.954	[−1.991; 2.927]
+1/NL [°]	0.258	0.058	Pearson	0.975	[−3.602; 4.546]
+1/SN [°]	0.238	0.171	Pearson	0.982	[−2.932; 4.324]
+1/NA [°]	0.050	0.057	Spearman	0.978	[−4.220; 3.189]
+1i/NA [mm]	0.551	0.347	Pearson	0.985	[−1.197; 0.985]
−1/MeGo [°]	0.086	0.056	Pearson	0.973	[−3.422; 5.182]
−1/NB [°]	0.173	0.261	Pearson	0.966	[−4.898; 3.510]
−1i/NB [mm]	0.297	0.089	Pearson	0.988	[−0.686; 0.798]
Interincisal angle [°]	0.058	0.018	Spearman	0.954	[−4.547; 6.831]

## 3 Results

This study included 379 patients, with 208 (54.88%) presenting SCIIMO and 171 (45.11%) with SCIIIMO. The total study collective consisted of patients aged between 5.3 and 53 years, with a mean age of 13 ± 6.7 years in class II and 14 ± 6.3 years in class III cases. Concerning the gender distribution, it was similar in both groups: among skeletal class II patients, 59% were female (*n* = 123), and in class III, 54% were female (*n* = 93). The characteristics of the study collective, including demographic and cephalometric data, are presented in Tables S2 and S3 for skeletal class II and III patients, respectively.

### 3.1 Machine‐Learning

**Figure 1:**
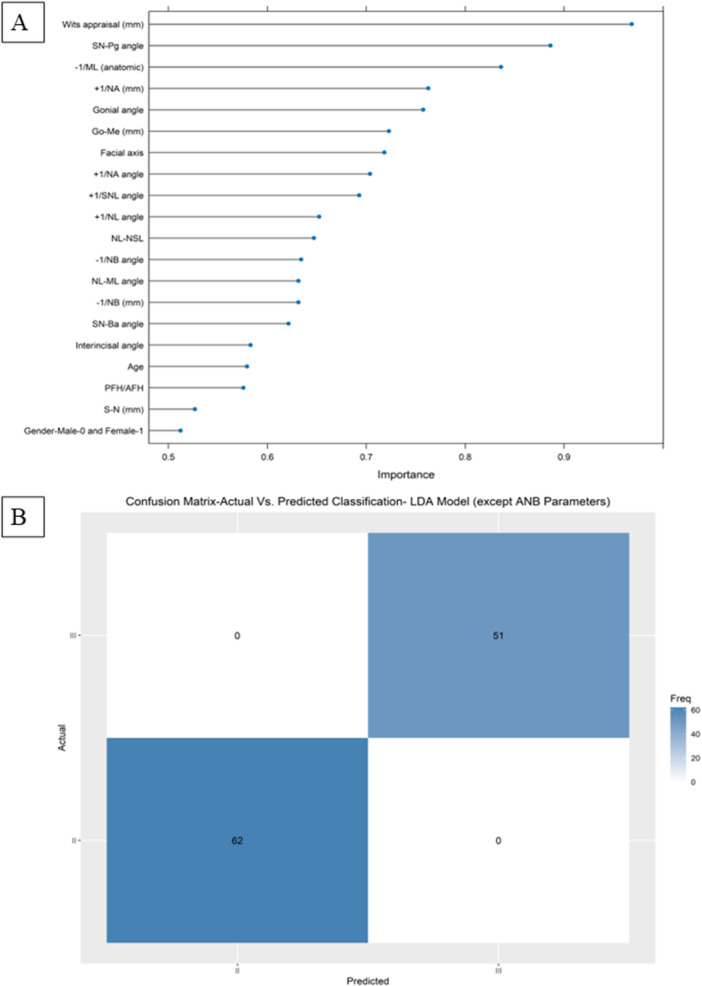
Importance of all cephalometric parameters, in addition to gender and age, while excluding the variables that are involved in computing the label (ANB, ANBind, Calculated_ANB, SNA, SNB, and ML‐NSL) to classify an individual as skeletal class II or III through machine learning (A) and the confusion matrix on the validation data (30% of the data) (B).

The following findings provide information about the performance of different machine‐learning Models in correctly determining an individual patient's skeletal class II or III. In the first tier (tier A), which was done as a sanity check only, we included the parameters SNA, SNB, ML‐NSL, and achieved 100% accuracy. In the next tier (B), we included all cephalometric parameters, in addition to gender and age, while excluding the variables which involved in computing the label (ANB, ANBind, Calculated_ANB, SNA, SNB, and ML‐NSL), and in this tier a mean accuracy of 100% was achieved in the LDA model followed by an accuracy of 97.74% in the RF model. Figure 1A presents the included cephalometric parameters, age, and gender, and their proportional importance in classifying an individual as a skeletal class II/III. Figure 1B shows the confusion matrix of the LDA model on the unseen data. As shown in Figure 1A, this tier was performed as a first step to examine the importance of the parameters and as a guide to the subsequent models (tiers C, D), which included all cephalometric variables, in addition to age and gender. Further models were conducted, with various numbers of cephalometric parameters included in the model to determine an individual's skeletal class II/III. The cephalometric parameters were chosen based on their chronological importance, as reported in Figure 1A. Hence, the tier C model included Wits appraisal only in the prediction, whereas the tier D model incorporated Wits appraisal and SN‐Pg angle. Table S4 demonstrates the performance of the tiers A‐D, although accuracy and kappa are reported for the best models only. It becomes evident that the inclusion of Wits‐appraisal only leads to high precision in determining skeletal class, that is, 94.72%. In contrast, the addition of the following important parameter, SN‐Pg, results in a slight increase in accuracy to 95.86% (accuracy of 0.958).

**Figure 2:**
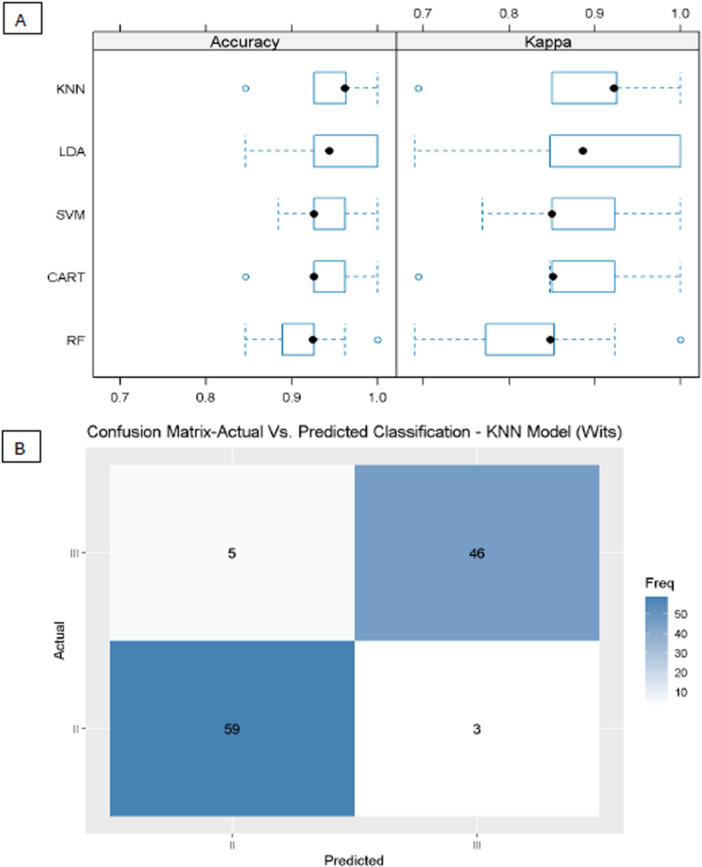
Accuracy and Kappa (A) and the confusion matrix on the validation data (30% of the data) (B) of model 1.

Comparing the different machine‐learning models (tier C), KNN, SVM, and LDA presented the highest mean accuracy, 94.72%, 94.37%, and 94.35%, respectively (Figure 2A). The confusion matrix also shows this high sensitivity and specificity (Figure 2B).

**Figure 3:**
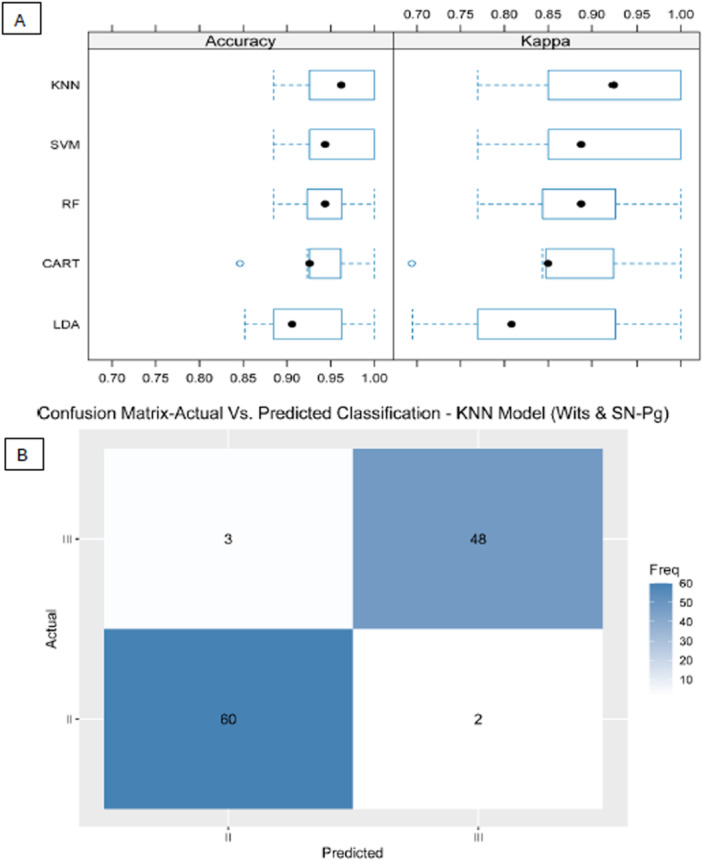
Accuracy and Kappa (A) and the confusion matrix on the validation data (30% of the data) (B) of model 2.

**Figure 4:**
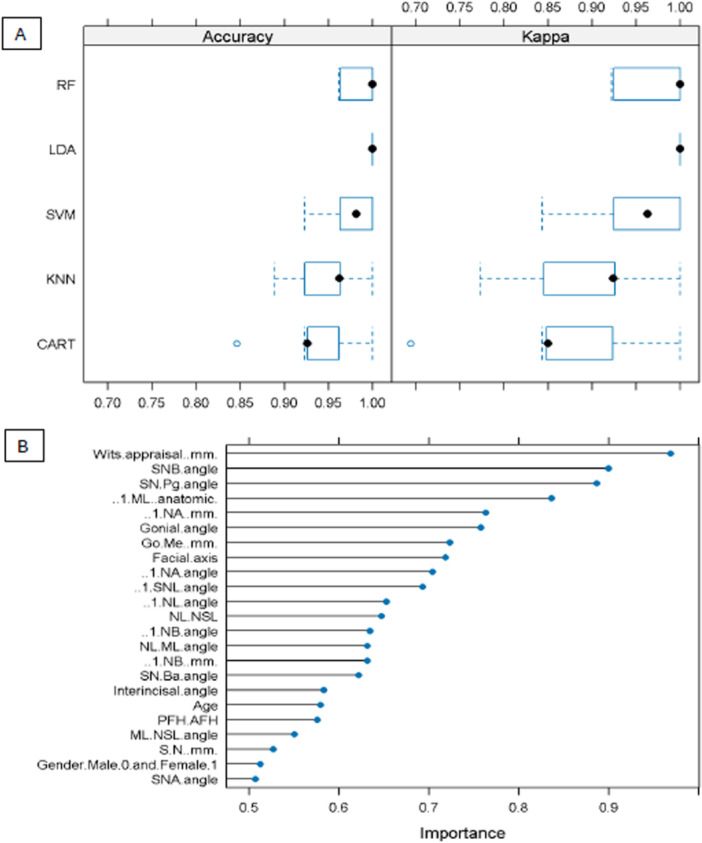
Accuracy and Kappa (A) and importance of the contributing factors (B) of model 3.

Consequently, tier D models were evaluated in terms of accuracy and reliability (kappa) (Figure 3A) and in terms of specificity and sensitivity (Figure 3B). In tier D, the best accuracy was reached by the machine‐learning models KNN and SVM, presenting a mean accuracy of 95.86% and 95.49%, respectively, followed by the models of RF, CART, and LDA, showing a mean accuracy of 93.98%, 93.23%, and 92.10%, respectively (Figure 3A). Finally, the confusion matrix of the models in tier D, presented in Figure 3B and 60 patients were correctly diagnosed as skeletal class III/II. Figure 4 shows the Accuracy and Kappa (A) and importance of the contributing factors (B) of model 3.

### 3.2 Clustering

**Figure 5:**
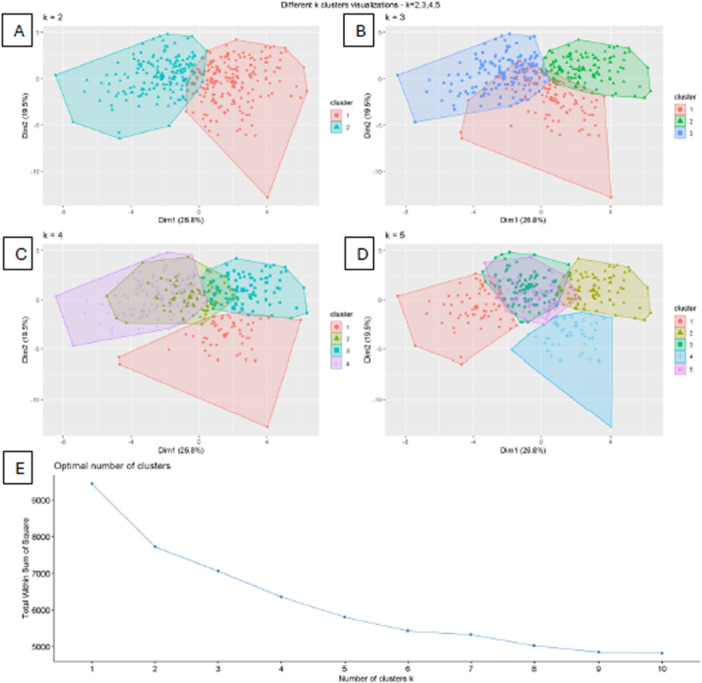
(A–D) show the cluster visualizations for different k clusters (*k* = 2, 3, 4, 5) numbers of clusters (k). In these figures, the X and Y axes represent dimensions 1 and 2. Finally, (E) represents the Elbow method to choose the optimal number of clusters (X‐axis number of clusters, while Y‐axis represents the Total within‐cluster sum of squares).

Initially, we included all parameters for the clustering process, and we started with evaluating the optimal cluster number using the Elbow method, which revealed *k* = 4 as the optimal number of clusters (Figure 5).

**Figure 6:**
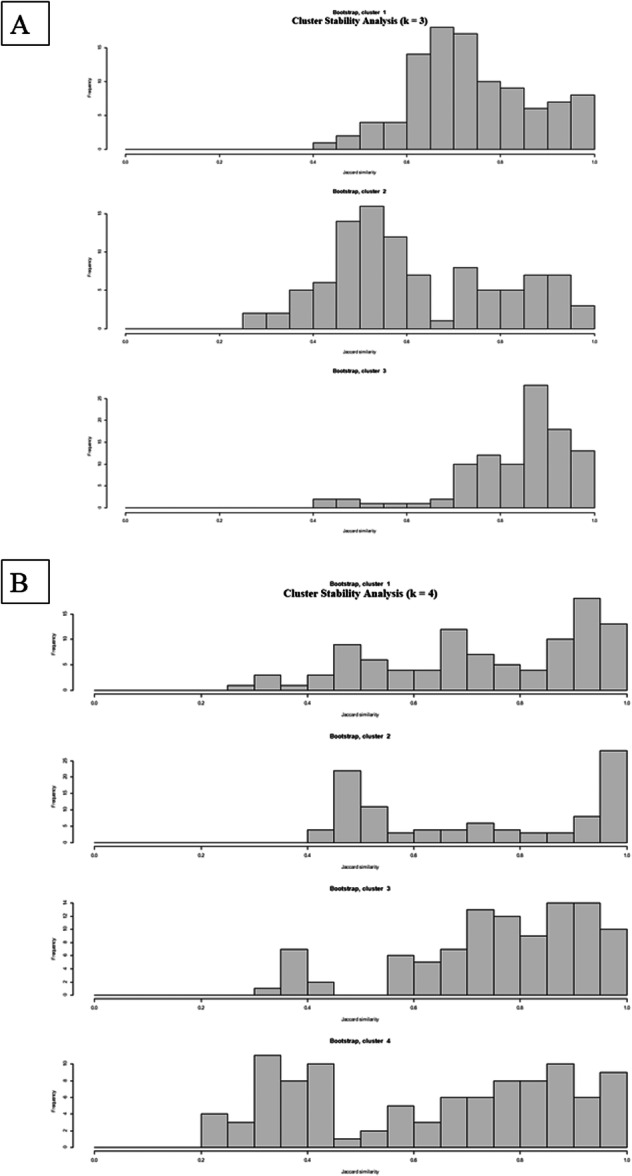
Presentation of the stability check of the clustering analysis when using *k* = 3 (A), and *k* = 4 (B), using the Jaccard similarity exam. The x‐axis represents the Jaccard similarity result, while the Y‐axis represents the frequency.

**Figure 7:**
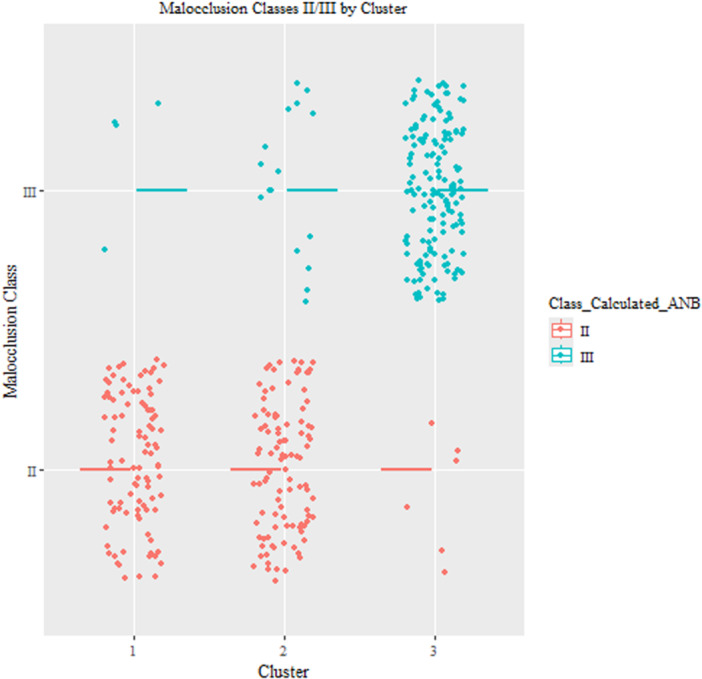
This figure shows the distribution of SCIIMO/SCIIIMO patients across the *k* = 3 clusters. The X‐axis represents the cluster, while the Y‐axis represents the malocclusion class.

**Figure 8:**
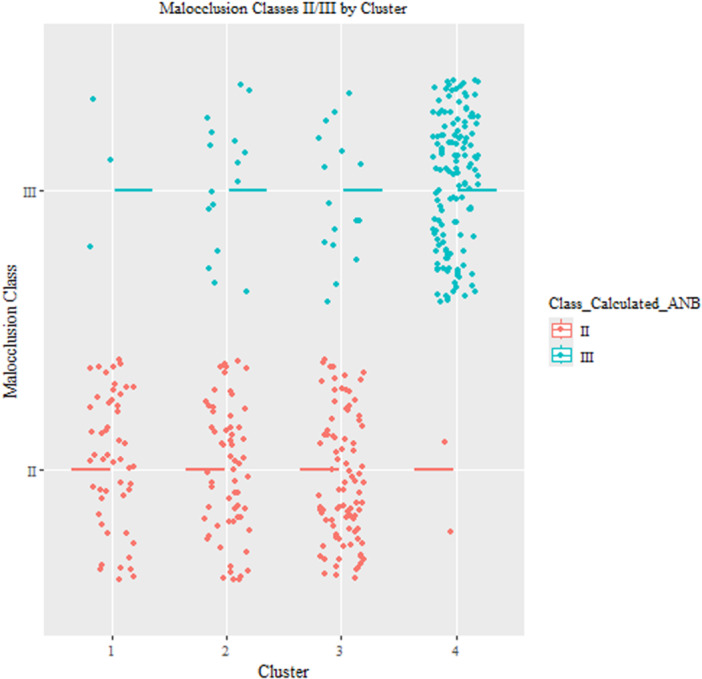
This figure shows the distribution of SCIIMO/SCIIIMO patients across the *k* = 4 clusters. The X‐axis represents the cluster, while the Y‐axis represents the malocclusion class.

Our analysis compared the results with *k* = 3 and *k* = 4 clusters. In Figure 6A,B, we presented the stability of the clusters when using *k* = 3 and *k* = 4. The results showed that when using *k* = 3, the clusters were more stable (mean Jaccard similarity clusters 1, 2, and 3 are 0.73, 0.62, and 0.83, respectively), compared to *k* = 4 (mean Jaccard similarity clusters 1, 2, 3, and 4 are 0.73, 0.72, 0.76, and 0.63, respectively). The results showed that when we have three clusters, SCIIMO patients were mainly found in the first two clusters (97%), while SCIIIMO patients were mainly available in the third cluster (88%). When analyzing the data using *k* = 4 clusters, the results also revealed that SCIIMO patients were primarily available in clusters 1–3, while SCIIIMO patients were available mainly in the fourth cluster (Table 2 and Figures 7, 8).

**Table 2:** K‐means clustering results summary when using all variables. Table 1 presents, for each clustering model, the number of patients in each cluster and their classification (SCIIMO/SCIIIMO).

Number of clusters	Cluster	Class Calculated ANB		Total
		II	III	
3 clusters	1	96	4	100
	2	106	16	122
	3	6	151	157
Total		208	171	379
4 clusters	1	51	3	54
	2	62	16	78
	3	93	16	109
	4	2	136	138
Total		208	171	379

In the next stage, we compared the cephalometric parameters differences between each cluster and found that in both options (i.e., *k* = 3, *k* = 4), there was a significant (*p* < 0.05) ANOVA result in all cephalometric parameters except S‐N (mm). For *k* = 3, noteworthy differences were seen between the clusters among gonial angles (higher in cluster three compared to the first cluster), ANB angle, Calculated_ANB, and Wits appraisal (lower in cluster three compared to the first two clusters). These results and others are described in Table 3. When we examined *k* = 4 clusters, we also revealed a significant (*p* < 0.05) difference between the four clusters except S‐N (mm) (Table 4). Finally, the age difference between the different clusters was not significant in both *k* = 3 and *k* = 4.

**Table 3:** The cephalometric parameters descriptive statistics mean (M) and standard deviation (SD) for each cluster among skeletal class II and III patients, when analyzing the data with three clusters. In addition, the tables present the ANOVA significance levels of the comparisons between the three clusters.

	1		2		3		
	M	SD	M	SD	M	SD	ANOVA
Age	13.46	6.51	12.97	6.70	14.02	6.37	NS
NL‐ML angle [°]	18.19	4.38	27.08	4.57	24.31	5.11	**
NL‐NSL angle [°]	7.41	3.55	9.36	3.23	6.71	2.95	**
PFH/AFH (%)	71.69	3.70	62.62	3.63	66.70	4.61	**
Gonial angle [°]	114.69	4.70	124.06	5.95	125.00	6.46	**
Facial axis	91.54	3.69	86.38	4.01	92.39	4.41	**
SNA angle [°]	81.94	3.51	79.88	3.15	81.50	3.69	**
SNB angle [°]	76.49	2.96	73.64	2.64	81.10	3.52	**
ANB angle [°]	5.46	1.97	6.24	2.40	0.40	2.45	**
ANB_ind_ [°]	2.74	1.39	4.08	1.23	3.65	1.41	**
Calculated_ANB (ANB – ANB_ind_) [°]	2.72	1.46	2.15	1.96	−3.24	1.95	**
SN‐Ba angle [°]	131.98	4.60	134.15	4.51	129.62	5.21	**
SN‐Pg angle [°]	78.21	2.80	74.29	2.58	82.16	3.63	**
S‐N (mm)	66.71	4.26	65.84	4.07	67.06	6.66	NS
Go‐Me (mm)	66.94	5.27	64.02	5.13	70.29	8.02	**
Wits appraisal (mm)	3.80	2.82	2.12	4.89	−4.42	4.52	**
ML‐NSL angle [°]	25.60	3.94	36.45	4.29	31.02	5.31	**
+1/NL angle [°]	72.38	12.37	70.81	7.20	64.06	6.98	**
+1/SNL angle [°]	79.79	12.73	80.17	7.80	70.76	7.01	**
+1/NA angle [°]	18.27	12.68	19.95	7.34	27.74	6.66	**
+1/NA (mm)	1.66	3.74	2.23	2.59	5.31	2.45	**
−1/ML (anatomic)	79.58	7.12	81.97	7.81	89.41	7.83	**
−1/NB angle [°]	22.52	7.53	28.13	6.59	22.71	7.45	**
−1/NB (mm)	3.00	2.14	5.26	2.30	3.12	2.35	**
Interincisal angle [°]	133.77	16.37	125.69	9.34	129.15	12.13	**

Abbreviation: NS, not significant.

* < 0.05.

** < 0.01.

**Table 4:** The cephalometric parameters descriptive statistics mean and standard deviation (SD) for each cluster among skeletal class II and III patients, when analyzing the data with four clusters. In addition, the tables present the ANOVA significance levels of the comparisons between the four clusters.

Cluster	1		2		3		4		
	M	SD	M	SD	M	SD	M	SD	ANOVA
Age	14.66	7.63	12.52	4.89	13.20	6.95	13.92	6.46	NS
NL‐ML angle [°]	17.90	4.09	19.96	4.72	27.51	4.60	24.76	5.03	**
NL‐NSL angle [°]	8.00	3.69	7.06	2.97	9.46	3.33	6.69	3.03	**
PFH/AFH (%)	71.51	4.19	70.35	3.89	62.18	3.50	66.34	4.54	**
Gonial angle [°]	114.41	4.63	117.03	5.61	124.42	6.03	125.79	6.10	**
Facial axis	91.11	3.33	91.31	4.91	86.33	3.76	92.37	4.38	**
SNA angle [°]	81.88	3.61	82.61	3.26	79.61	3.12	81.11	3.64	**
SNB angle [°]	76.18	3.41	77.67	3.32	73.47	2.62	81.06	3.59	**
ANB angle [°]	5.70	2.20	4.95	2.11	6.14	2.49	0.05	2.30	**
ANB_ind_ [°]	2.77	1.36	3.29	1.50	4.08	1.25	3.57	1.42	**
Calculated_ANB (ANB – ANB_ind_) [°]	2.93	1.77	1.66	2.02	2.06	2.00	−3.52	1.83	**
SN‐Ba angle [°]	131.60	4.53	132.19	4.73	134.32	4.55	129.40	5.17	**
SN‐Pg angle [°]	78.01	3.35	78.91	3.08	74.11	2.58	82.15	3.72	**
S‐N (mm)	67.38	4.45	65.60	4.01	65.86	3.89	67.38	6.96	*
Go‐Me (mm)	67.91	5.30	66.38	6.19	64.08	5.02	70.36	8.11	**
Wits appraisal (mm)	4.09	3.10	1.98	4.69	2.25	4.14	−4.89	4.53	**
ML‐NSL angle [°]	25.91	4.57	27.02	4.22	36.97	4.18	31.45	5.26	**
+1/NL angle [°]	81.77	7.59	60.98	6.81	71.67	6.57	64.85	6.69	**
+1/SNL angle [°]	89.79	7.97	68.03	6.63	81.13	6.89	71.53	6.75	**
+1/NA angle [°]	8.35	7.63	29.35	6.79	19.26	6.68	27.36	6.57	**
+1/NA (mm)	−1.14	2.69	5.02	2.08	2.06	2.44	5.20	2.49	**
−1/ML (anatomic)	81.36	7.71	77.21	5.58	82.84	7.53	90.94	6.93	**
−1/NB angle [°]	20.73	8.48	27.49	6.42	27.61	6.62	21.56	6.94	**
−1/NB (mm)	2.39	2.32	4.43	2.09	5.17	2.28	2.85	2.26	**
Interincisal angle [°]	145.24	12.51	118.22	8.00	126.99	8.83	131.03	11.36	**

Abbreviation: NS, not significant.

* < 0.05.

** < 0.01.

## 4 Discussion

The present study aimed to deepen our understanding and analyze the clustering of skeletal class II/III German patients, the diversity of malocclusion, and to establish a machine‐learning model to classify them as skeletal class II/III cases correctly, with a reduced set of readily available cephalometric parameters. Testing various machine‐learning models with different input variables revealed that model 1, which incorporated only the parameter Wits appraisal, led to an accuracy of 94.72% and a kappa of 89.7%. The addition of the following important parameter, SN‐Pg, results in a slight increase in accuracy to 95.86%. Due to these high accuracy values, adding further variables, including cephalometrics, age, and gender, did not result in a relevant increase in the model's precision, suggesting that the Wits appraisal only provides sufficient information for the machine‐learning model classification.

Zhou et al. (2023) compared nine machine‐learning models intended to make cephalometric diagnoses in both the sagittal and vertical directions. Generally, the mean accuracy of the machine‐learning models was higher in the sagittal (91.60% ± 5.43%) than in the vertical direction (82.25% ± 6.37%). Among the different models tested, the best fit presented the multi‐layer perceptron (97.56%) and the linear support vector machine (90.24%) in the sagittal and vertical planes. Another study, which applied the machine‐learning model Support Vector Machines to correctly diagnose Colombian individuals as skeletal classes I, II, and III, achieved an accuracy of 74.51%. Contrary to our study, they used craniomaxillary cephalometric variables only. Here, the extension to mandibular cephalometric parameters and the testing of various machine‐learning models in our study might explain the higher accuracy (94.9%) (Niño‐Sandoval et al. 2016).

The exploratory clustering analysis revealed the craniofacial phenotype diversity for each cluster within all data and for each group (SCIIMO/SCIIIMO) based on various cephalometric parameters, like Gonial angle, ANB angle, Calculated_ANB, and Wits appraisal. In both *k* = 3 and *k* = 4, the last cluster mainly consisted of SCIIIMO patients, while the other clusters consisted of SCIIMO patients. And this division was consistent with the means of the cephalometric parameters within each cluster (Tables 3 and 4). Finally, the cluster analysis for age was not significant between the clusters.

A study that was done by Alshoaibi et al. (2023) on skeletal class III malocclusion in adult Chinese patients identified the number of skeletal patterns by a clustering algorithm performed separately from three to seven clusters depending on the 76 3D measurements. This study found that the second cluster showed that the severe skeletal class III contained only 11.4% of cases with a maxillary deficiency and severe mandibular prognathism (Alshoaibi et al. 2023). Another study was done by Moreno Uribe et al. (2014) about phenotypic diversity in white adults with Class II malocclusion, which found that cluster analysis identified five distinct Class II phenotypes. However, models with 2, 3, or 4 clusters were also statistically acceptable (Moreno Uribe et al. 2014).

## 5 Limitations

A limitation might be that patients with skeletal class I cannot be correctly classified using this study's methods. However, since the investigation was intended to compare only class II/III patients, this cannot be regarded as a significant limitation; instead, it calls for future studies, including skeletal class I participants. While the results revealed in this study are promising, we should take the models presented with cautious interpretation, due to the lack of external validation, and future studies should use additional cohorts that will take into account additional factors, like other ethnic groups. In addition to the broad range of age that were included in this study, and although we included gender and age as covariates in the first tier, future studies should validate that this study can diagnose patients with different age groups. Besides, in this study, we've used machine learning models that might have the potential for leakage for the target variable. However, these models were not considered novel, and were done as an exploratory tool to understand the importance of the parameters, in order to proceed to the next models. In addition, the typical classification methods' accuracy is limited, and many times it needs the orthodontist's further assessment. While here, the machine learning models were able to classify these patients more accurately. Finally, clustering analysis was done as an exploratory analysis that revealed unique clusters in our sample.

## 6 Conclusion

Overall, our study encourages more research that aims to examine and validate these study models, and additional models, that will lead to a practical, simple, and precise classification of the skeletal malocclusion of an individual patient, which should increase not only the diagnostic precision but also reduce false treatment planning in orthodontics. In this study, the Wits appraisal captures much of the same sagittal discriminatory information as the predefined class construct, which explains its crucial importance in the presented models. In the exploratory clustering analysis, three or four distinct clusters emerged as optimal when analyzing the differences among SCIIMO/SCIIIMO patients, emphasizing the complexity and diversity of malocclusion phenotype.

## Author Contributions

Eva Paddenberg‐Schubert contributed to design, data acquisition, analysis, and drafted the manuscript. Kareem Midlej contributed to design, data acquisition, analysis, and drafted the manuscript. Sebastian Krohn contributed to design, data acquisition, analysis, and drafted the manuscript. Iqbal M. Lone contributed to design, data analysis, and drafted the manuscript. Osayd Zohud contributed to design, data analysis, and critically revised the manuscript. Obaida Awadi contributed to design, data analysis, and critically revised the manuscript. Samir Masarwa contributed to design, data analysis, and critically revised the manuscript. Aysar Nashef contributed to design, data analysis, and critically revised the manuscript. Christian Kirschneck contributed to design, data acquisition, analysis, and critically revised the manuscript. Nezar Watted contributed to conception, data acquisition, analysis, and critically revised the manuscript. Peter Proff contributed to conception and design, data acquisition, and critically revised the manuscript. Fuad A. Iraqi contributed to conception and design, data acquisition, analysis, and interpretation, and critically revised the manuscript. All authors gave their final approval and agreed to be accountable for all aspects of the work.

## Ethics Statement

Before the collection of the samples, ethical approval of the corresponding committee of the University of Regensburg was granted for this project (approval number 19‐1596‐101, 13/11/2019).

## Consent

Informed written consent was obtained from all subjects involved in the study.

## Conflicts of Interest

The authors declare no conflicts of interest.

## Supporting information

Supporting File 1

Supporting File 2

Supporting File 3

Supporting File 4

Supporting File 5

## Data Availability

The data are available with the corresponding author and can be accessed with a reasonable request, and according to the ethical committee principles.
