# Supplementary figures and images for: Machine Learning and Clustering Analysis of Class II and III Malocclusions
Source: Clin Exp Dent Res. 2026 Jun 1;12(3):e70384. doi: 10.1002/cre2.70384 (PMC13239717; doi:10.1002/cre2.70384)

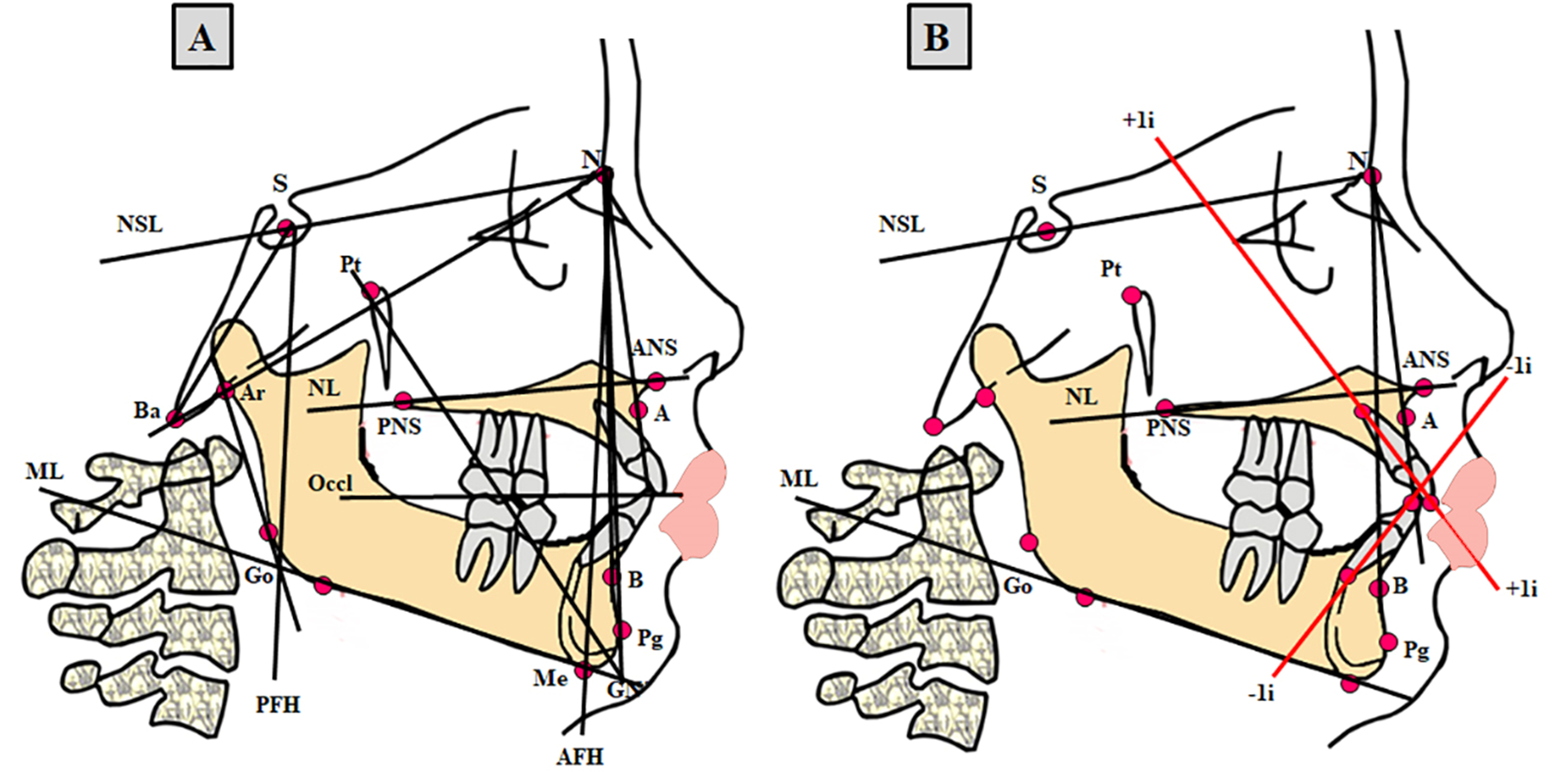

Supplement: Supplementary file 1 — Supporting File 1 [file CRE2-12-e70384-s002.tif]
