# Supplementary material for: Machine Learning and Clustering Analysis of Class II and III Malocclusions
Source: Clin Exp Dent Res. 2026 Jun 1;12(3):e70384. doi: 10.1002/cre2.70384 (PMC13239717; doi:10.1002/cre2.70384)
Supplement: Supplementary file 2 — Supporting File 2 [file CRE2-12-e70384-s001.docx]

**Supplement Table S1.**

|  | parameter | definition | Figure 1 |
| --- | --- | --- | --- |
|  |  |  | Figure 1A |
| Sagittal | SNA [°] | angle between Sella, Nasion, and point A |  |
|  | SNB [°] | angle between Sella, Nasion, and point B |  |
|  | ANB [°] | angle between Nasion, point A, and point B |  |
|  | Wits [mm] | sagittal distance between A’ and B’ at Occl. Negative, if B’ is anterior to A’ and positive, if B’ is posterior to A’ |  |
|  | SN-Ba [°] | angle between Sella, Nasion and Basion |  |
|  | SN-Pg [°] | angle between Sella, Nasion and Pogonion |  |
|  | S-N [mm] | length of the anterior cranial base, distance between Sella and Nasion |  |
|  | Go-Me [mm] | length of the mandibular plane, distance between Go and Me |  |
| Vertical | NL/NSL | angle between Sella-Nasion-line (NSL = SN) and nasal line (Spa-Spp) |  |
|  | NL/ML | angle between the NL and ML |  |
|  | PFH/AFH | ratio between posterior (SGo) and anterior (NMe) facial height |  |
|  | Gonion angle | angle between ML and line GoAr at Gonion |  |
|  | Facial axis | angle between the lines NBa and PtGN’ |  |
|  | ML-NSL angle | the angle formed between the ML and NSL lines |  |
|  |  |  | Figure 1B |
| Dental  Dental | +1/NL [°] | angle between upper incisors’ tooth axis and line NL |  |
|  | +1/SN [°] | angle between upper incisors’ tooth axis and line SN |  |
|  | +1/NA [°] | angle between upper incisors’ tooth axis and line NA |  |
|  | +1i/NA [mm] | perpendicular distance between line NA and upper incisal point |  |
|  | -1/MeGo [°] | angle between lower incisors’ tooth axis and line ML |  |
|  | -1/NB [°] | angle between lower incisors’ tooth axis and line NB |  |
|  | -1i/NB [mm] | perpendicular distance between line NB and the lower incisal point |  |
|  | Interincisal_angle [°] | angle between tooth axes of the lower and upper incisors |  |
