# Supplementary material for: Machine Learning and Clustering Analysis of Class II and III Malocclusions
Source: Clin Exp Dent Res. 2026 Jun 1;12(3):e70384. doi: 10.1002/cre2.70384 (PMC13239717; doi:10.1002/cre2.70384)
Supplement: Supplementary file 3 — Supporting File 3 [file CRE2-12-e70384-s003.docx]

**Supplement Table S2.**

| Class II |  | | | | | | |
| --- | --- | --- | --- | --- | --- | --- | --- |
| Variable | N | M | Std. Dev. | Min | Pctl. 25 | Pctl. 75 | Max |
| Age | 208 | 13 | 6.7 | 5.4 | 10 | 14 | 53 |
| 0<Age<13 | 162 (78%) |  |  |  |  |  |  |
| 14<Age<20 | 33 (16%) |  |  |  |  |  |  |
| Age>21 | 13 (6%) |  |  |  |  |  |  |
| Female | 123 (59%) |  |  |  |  |  |  |
| Male | 85 (41%) |  |  |  |  |  |  |
| NL-ML angle [°] | 208 | 23 | 6.1 | 4 | 19 | 27 | 42 |
| NL-NSL angle [°] | 208 | 8.4 | 3.4 | -1.8 | 6.1 | 10 | 19 |
| PFH/AFH (%) | 208 | 67 | 5.3 | 54 | 63 | 71 | 85 |
| Gonial angle [°] | 208 | 119 | 6.9 | 104 | 114 | 124 | 139 |
| Facial axis | 208 | 89 | 4.4 | 71 | 86 | 91 | 99 |
| SNA angle [°] | 208 | 81 | 3.2 | 73 | 79 | 83 | 89 |
| SNB angle [°] | 208 | 75 | 2.9 | 66 | 73 | 77 | 82 |
| ANB angle [°] | 208 | 6.3 | 1.6 | 1.7 | 5.2 | 7.4 | 12 |
| ANB_ind_ [°] | 208 | 3.5 | 1.4 | -0.46 | 2.6 | 4.5 | 8.1 |
| Calculated_ANB (ANB – ANB_ind_) [°] | 208 | 2.8 | 1.1 | 1.5 | 2 | 3.4 | 8.6 |
| SN-Ba angle [°] | 208 | 133 | 4.6 | 123 | 130 | 137 | 145 |
| SN-Pg angle [°] | 208 | 76 | 3.1 | 67 | 74 | 78 | 85 |
| S-N (mm) | 208 | 66 | 5 | 57 | 63 | 69 | 107 |
| Go-Me (mm) | 208 | 65 | 5.9 | 54 | 61 | 68 | 100 |
| Wits appraisal (mm) | 208 | 3.3 | 4.1 | -28 | 1.9 | 5.3 | 13 |
| ML-NSL angle [°] | 208 | 31 | 6.3 | 13 | 27 | 35 | 47 |
| +1/NL angle [°] | 208 | 71 | 10 | 46 | 64 | 78 | 113 |
| +1/SNL angle [°] | 208 | 80 | 11 | 53 | 73 | 87 | 122 |
| +1/NA angle [°] | 208 | 19 | 11 | -22 | 12 | 26 | 47 |
| +1/NA (mm) | 208 | 1.9 | 3.2 | -12 | 0.075 | 4.3 | 10 |
| -1/ML (anatomic) | 208 | 80 | 7.2 | 58 | 76 | 84 | 104 |
| -1/NB angle [°] | 208 | 26 | 7.5 | 4 | 21 | 31 | 41 |
| -1/NB (mm) | 208 | 4.3 | 2.5 | -2.5 | 2.6 | 5.7 | 12 |
| Interincisal angle [°] | 208 | 129 | 14 | 89 | 119 | 135 | 187 |
