# Supplementary material for: Machine Learning and Clustering Analysis of Class II and III Malocclusions
Source: Clin Exp Dent Res. 2026 Jun 1;12(3):e70384. doi: 10.1002/cre2.70384 (PMC13239717; doi:10.1002/cre2.70384)
Supplement: Supplementary file 4 — Supporting File 4 [file CRE2-12-e70384-s004.docx]

**Supplementary Table S3.**

| Class III |  | | | | | | |
| --- | --- | --- | --- | --- | --- | --- | --- |
| Variable | N | M | Std. Dev. | Min | Pctl. 25 | Pctl. 75 | Max |
| Age | 171 | 14 | 6.3 | 5.3 | 11 | 15 | 49 |
| 0<Age<13 | 120 (70%) |  |  |  |  |  |  |
| 14<Age<20 | 31 (18%) |  |  |  |  |  |  |
| Age>21 | 20 (12%) |  |  |  |  |  |  |
| Female | 93 (54%) |  |  |  |  |  |  |
| Male | 78 (46%) |  |  |  |  |  |  |
| NL-ML angle [°] | 171 | 25 | 5.4 | 11 | 21 | 28 | 40 |
| NL-NSL angle [°] | 171 | 6.9 | 3.2 | -1.5 | 4.8 | 9.1 | 16 |
| PFH/AFH (%) | 171 | 66 | 5.3 | 54 | 63 | 70 | 84 |
| Gonial angle [°] | 171 | 125 | 6.5 | 103 | 122 | 129 | 142 |
| Facial axis | 171 | 92 | 4.7 | 80 | 89 | 95 | 105 |
| SNA angle [°] | 171 | 81 | 3.9 | 70 | 79 | 84 | 91 |
| SNB angle [°] | 171 | 81 | 3.7 | 71 | 78 | 83 | 92 |
| ANB angle [°] | 171 | 0.32 | 2.3 | -10 | -0.8 | 1.9 | 4.9 |
| ANB_ind_ [°] | 171 | 3.6 | 1.5 | -0.72 | 2.7 | 4.7 | 7.2 |
| Calculated_ANB (ANB – ANB_ind_) [°] | 171 | -3.3 | 1.7 | -13 | -4 | -2.1 | -1.5 |
| SN-Ba angle [°] | 171 | 130 | 5.4 | 110 | 127 | 134 | 144 |
| SN-Pg angle [°] | 171 | 82 | 3.9 | 72 | 79 | 84 | 94 |
| S-N (mm) | 171 | 67 | 5.7 | 56 | 64 | 68 | 116 |
| Go-Me (mm) | 171 | 70 | 7.4 | 54 | 66 | 73 | 123 |
| Wits appraisal (mm) | 171 | -4.4 | 4.1 | -41 | -5.6 | -2.2 | 3 |
| ML-NSL angle [°] | 171 | 31 | 6.2 | 14 | 27 | 36 | 48 |
| +1/NL angle [°] | 171 | 65 | 7.2 | 46 | 61 | 69 | 85 |
| +1/SNL angle [°] | 171 | 72 | 7.4 | 53 | 67 | 78 | 90 |
| +1/NA angle [°] | 171 | 27 | 6.9 | 8.3 | 22 | 32 | 44 |
| +1/NA (mm) | 171 | 5.1 | 2.5 | -1.3 | 3.6 | 6.5 | 14 |
| -1/ML (anatomic) | 171 | 90 | 7.6 | 68 | 85 | 94 | 111 |
| -1/NB angle [°] | 171 | 23 | 7.5 | 2.5 | 18 | 28 | 42 |
| -1/NB (mm) | 171 | 3.2 | 2.4 | -2.8 | 1.7 | 4.9 | 11 |
| Interincisal angle [°] | 171 | 130 | 12 | 94 | 122 | 138 | 162 |
