# Supplementary material for: Machine Learning and Clustering Analysis of Class II and III Malocclusions
Source: Clin Exp Dent Res. 2026 Jun 1;12(3):e70384. doi: 10.1002/cre2.70384 (PMC13239717; doi:10.1002/cre2.70384)
Supplement: Supplementary file 5 — Supporting File 5 [file CRE2-12-e70384-s005.docx]

**Supplementary Table S4.**

| **Model** | **Tier** | **Best model** | **Hyperparameters** | **Accuracy** | **Kappa** |
| --- | --- | --- | --- | --- | --- |
| **Model 1 – SNA, SNB, ML-NSL Angles** | **A** | **LDA, KNN, SVM** | **KNN- k=5**  **SVM- sigma= 0.62 and C = 0.25** | **100%** | **100%** |
| **All parameters except for ANB, ANBind, Calculated_ANB, SNA, SNB, and ML-NSL Angles** | **B** | **LDA** |  | **100%** | **100%** |
| **Model 1: Wits appraisal** | **C** | **KNN** | **KNN – k=7** | **94.72%** | **89.39%** |
| **Model 1: Wits appraisal (with noise)** | **C** | **CART** |  | **93.60%** | **87.06%** |
| **Model 2: Wits appraisal + SN-Pg angle** | **D** | **KNN** | **KNN - k=5** | **95.86%** | **91.66%** |
| **Model 2: Wits appraisal + SN-Pg angle (with noise)** | **D** | **SVM** | **SVM - sigma = 1.51 and C = 1** | **95.88%** | **91.68** |
